# Memory for novel and familiar environments relies on the hippocampus: A case study on a patient with a right anteromesial temporal lobectomy

**DOI:** 10.1002/hipo.23086

**Published:** 2019-03-08

**Authors:** Michiel H. G. Claessen, Martine J. E. van Zandvoort, Frans S. S. Leijten, Ineke J. M. van der Ham

**Affiliations:** ^1^ Experimental Psychology, Helmholtz Institute Utrecht University Utrecht the Netherlands; ^2^ Centre of Excellence in Rehabilitation Medicine Brain Centre Rudolf Magnus, University Medical Centre Utrecht, and De Hoogstraat Rehabilitation Utrecht the Netherlands; ^3^ Department of Health, Medical and Neuropsychology Leiden University Leiden the Netherlands; ^4^ Department of Neurology and Neurosurgery Rudolf Magnus Institute, University Medical Centre Utrecht Utrecht the Netherlands

**Keywords:** epilepsy, hippocampus, neurosurgery, spatial memory, spatial navigation

## Abstract

While the hippocampus has been ascribed a prominent role in navigation ability, it is still a subject of debate whether it contributes to learning novel environments only or to remembering familiar environments as well. We attempt to shed light on this issue by reporting on a patient who developed complaints of severe difficulties with navigation after she underwent a right anteromesial temporal lobectomy. A standard neuropsychological assessment revealed only a visuospatial working memory deficit. Clear evidence for problems with novel environments were found on a virtual route learning test. Two real‐world tests were used to investigate her ability to recall familiar environments. The first test was based on the area she grew up in (and still visits regularly) and the second test concerned her current place of residence which she never visited prior to the surgery. While her landmark recognition in general was accurate, she showed notable difficulties with indicating their locations on a map and with giving accurate route descriptions between them for both real‐world environments. This pattern of findings suggests that the hippocampus is not only important for navigation in novel environments, but also for familiar environments learned long ago.

## 1 INTRODUCTION

In his Nobel Prize winning research on rodents, John O'Keefe pointed out the relationship between the hippocampus and spatial memory, particularly the ability to create environmental cognitive maps (O'Keefe & Nadel, 1978). This is an important element of navigation ability (Schinazi, Nardi, Newcombe, Shipley, & Epstein, 2013). This relationship has also been found in human research. For example, temporal lobectomy patients, who have undergone surgical removal of the (right) hippocampus and adjacent temporal lobe structures for relief of intractable epilepsy, perform worse on navigation tasks than healthy controls (Astur, Taylor, Mamelak, Philpott, & Sutherland, 2002; Maguire, Burke, Phillips, & Staunton, 1996; Spiers et al., 2001). Given these results, it is rather striking that currently only one article has provided detailed case reports on temporal lobectomy patients with navigation impairment (Iaria et al., 2016).

Another ongoing debate in the literature concerns the precise role the hippocampus plays in navigation. Some case studies have suggested that its contribution to navigation is time‐limited, given that these patients had difficulties only in novel and not familiar environments (Rusconi, Morganti, & Paladino, 2008; Teng & Squire, 1999). However, several other case studies have reported on patients who are hindered by navigation problems in both novel and familiar environments. This suggests that the involvement of the hippocampus in navigation is of permanent nature (Maguire, Nannery, & Spiers, 2006; Rosenbaum et al., 2000; Rosenbaum, Gao, Richards, Black, & Moscovitch, 2005). Hence, current findings with regard to this issue are highly inconsistent.

The contribution of the current study is twofold. First, it is among the first to provide a detailed case report on a patient with serious navigation difficulties after a right anteromesial temporal lobectomy. This suggests that this is a rarely described complication after such an intervention. Second, it contributes to the ongoing debate about the specific nature of the hippocampal involvement in navigation.

**Figure 1:**
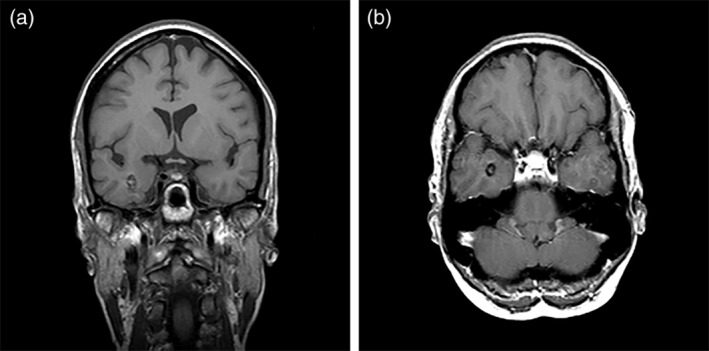
MRI scans taken 1 year before surgery (a: coronal view, b: axial view). Note the location of the cavernous hemangioma (measuring 9 mm in diameter) in the right medial temporal lobe directly lateral to the head of the hippocampus. There was no sign of hippocampal sclerosis. The right side of the brain corresponds with the left side

**Figure 2:**
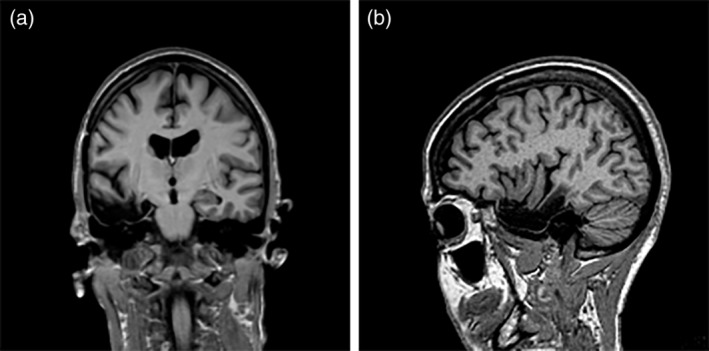
MRI scans, taken 15 months after surgery, show the resection size (i.e., 6 cm from the anterior temporal pole in posterior direction, 5 cm on the left–right axis, and 2 cm on the dorsoventral axis). The hippocampus head, body, and part of the tail were resected. The anterior part of the amygdala was spared. All but the anterior and medial rim of the amygdala turned into gliotic tissue, possibly due to ischemia. Furthermore, the anterior part of parahippocampal gyrus (leaving a small medial rim), the anterior part of the fusiform gyrus, the anterior inferior part of the superior temporal gyrus, the anterior part of the middle temporal gyrus, and the inferior temporal gyrus were also resected. This includes the entorhinal and transentorhinal region. (a) Coronal view, (b) sagittal view. Later scans (4 and 12 years after surgery) showed that the lesion area has not changed over time; nor were there any indications of atrophy or other brain pathology. The right side of the brain in the coronal image corresponds with the left side

This case study concerns Z.R., a right‐handed, 66‐year‐old woman diagnosed with epilepsy due to a right mesiotemporal cavernous hemangioma at the age of 44 (see Figure 1). Until then, she had been the keystone of a family with five children. Her seizures (partial and generalized seizures, and absences) were associated with postictal complaints of spatial disorientation, sometimes lasting up to 3 days. The seizures gradually increased in frequency and severity, and medication turned out to be ineffective in controlling the seizures. After a few years, her husband asked for a divorce, as he found himself unable to cope with the situation. Ten years after the onset of the seizures, Z.R. lost her job as she had been on sick leave for a long period of time due to a series of severe seizures. At the age of 54, she underwent epilepsy surgery with a right anteromesial temporal lobectomy that included amygdalohippocampectomy, and lesionectomy (see Figure 2). Except for around five brief auras a year, she has since been seizure‐free and epileptic medication has been completely tapered off.

Almost 12 years postsurgery, she was brought to our attention and suffered from severe navigation problems. Although she had consulted others, no convincing explanation was provided based on earlier investigations. She stated that she has never been a very skilled navigator. However, apart from postictal complaints of spatial disorientation, it was only after the surgery that these problems started to severely affect her daily life functioning. Her impression was that her navigation problems particularly concern environments she has first encountered after the surgery. To cope with this, she records elaborate written route descriptions in a notebook as she finds herself unable to learn new routes no matter how many times she has travelled them. She relies rigidly on specific landmarks (e.g., a mailbox) to find her way around, and gets confused when things are only slightly different from expected (e.g., when the design of a shop display has been changed). Consequently, she currently lives in an apartment owned by a health facility organization for people with acquired brain injury that provides continuous support. She also reports some memory complaints (e.g., forgetting that she had put the kettle on to make tea when she leaves the room in the meantime) and severe fatigue.

Z.R. has a medical history of traumatic brain injury following collision with a car at the age of seven; further details concerning this injury are unknown. Her psychiatric history specifies multiple depressive and dysthymic episodes (with two periods of hospitalization) and a diagnosis of personality disorder not otherwise specified (DSM‐IV) based on an enduring and stable pattern of difficulties with establishing and maintaining social relationships. Z.R. has suffered from episodes of depression and personality issues for a large part of her life starting many years before the onset of the epileptic seizures. A causal connection between her depressive episodes and her navigation problems is not likely, as such specific cognitive impairments are uncommon in depressed patients (Lezak, Howieson, Bigler, & Tranel, 2012).

Neuropsychological assessments were performed (not by us) before surgery, briefly after surgery, and 1 and 3 years postsurgery. In the report of the presurgery assessment, it was indicated that her intellectual functioning is above average with a minor discrepancy between verbal (above average) and visual abilities (high average), likely related to the right‐sided cavernous hemangioma. Her performance was above average on tests for language, attention and concentration, and mental flexibility. Visuospatial test performance fell in the average range. No memory problems were found. The only remarkable finding was that she tended to approach complex tasks using a trial‐and‐error strategy. The reports of all postsurgery assessments stated that performance patterns were identical to that in the presurgery assessment, and did not provide a solid explanation for her profound navigation problems.

We started our investigation with a comprehensive neuropsychological assessment to verify her current cognitive status (almost 12 years postsurgery). We found (above) average performance on all tests, but she performed low on a visuospatial working memory task (Corsi Block‐Tapping Task; see Table 1) indicating a deficit in manipulating visuospatial information.

**Table 1:** Z.R.'s performance on our neuropsychological assessment nearly 12 years after the surgery

Cognitive domain	Test	Raw scores	Interpretation
General cognitive functioning	Cognitive screening test	CST‐14:14	Unimpaired
		CST‐20:20	Unimpaired
	National adult reading test	93 (estimated IQ = 123)	Above average
Language	Boston naming test	84/87 (171/177)	80th percentile
Working memory	Digit span (WAIS‐IV)	34 (SS = 16)	Above average
	Forward score/span	12/8	
	Backward score/span	12/6	
	Sorting score/span	10/6	
	Corsi block‐tapping task		
	Forward score/span	9/6	70th percentile
	Backward score/span	4/3	5th–10th percentile
Memory	Rey auditory verbal learning task		
	Immediate recall	52 (5/9/10/14/14)	84th percentile
	Delayed recall	13/15	95th percentile
	Delayed recognition	29/30 (1 miss)	Unimpaired
	Rivermead behavioral memory test—story		
	Immediate recall	24.5	88th percentile
	Delayed recall	21.5	86th percentile
	% retained	88%	62th percentile
	Rey complex figure		
	Delayed recall (30 minutes)	16/36	> 50th percentile
	Location learning test		
	Displacement score	8 (5/3/0/0/×)	60‐70th percentile
	Learning index	0.85	70th percentile
	Delayed recall score	0	> 75th percentile
	Benton visual retention test		
	Version C	6/10	Unimpaired
Attention/speed	Star cancellation (BIT)	70 s; systematic working method, from left to right	Unimpaired
	Color word interference test (D‐KEFS)		
	Condition 1 (color naming)	30 s (GS = 11)	Average
	Condition 2 (word reading)	25 s (GS = 10)	Average
Executive functioning	Color word interference test (D‐KEFS)		
	Condition 3 (inhibition)	44 s (GS = 14)	Above average
	Condition 4 (inhibition and switching)	48 s (GS = 15)	Above average
Visual perception	Cortical vision screening test		
	Symbol acuity	36/36	Unimpaired
	Shape discrimination	8/8	Unimpaired
	Size discrimination	2/2	Unimpaired
	Shape detection	8/8	Unimpaired
	Hue detection	4/4	Unimpaired
	Dot counting	4/4	Unimpaired
	Fragmented numbers	8/8	Unimpaired
	Face perception	8/8	Unimpaired
	Crowding test	4/4	Unimpaired
	Birmingham object recognition battery		
	Size match task	29/30	Unimpaired
	Length match task	27/30	Unimpaired
	Judgment of line orientation	30/30	> 86th percentile
	Benton facial recognition test	54/54	> 98th percentile
Visuoconstruction	Rey complex figure		
	Direct copy	34/36	> 50th percentile
Spatial abilities	Road map test (mental rotation)	90 s (1 error)	Unimpaired
	Bergen left–right discrimination test		
	Condition 1 (back)	31 (2 errors)	Unimpaired
	Condition 2 (front)	33 (1 error)	Unimpaired
	Condition 3 (mixed)	37	Unimpaired

*Note*. Corrections for sex, age, and education level have been applied to the raw scores if available. Percentile scores are displayed for all tests that allow for calculation of this type of score. Scaled scores (SS and GS) have a mean of 10 and standard deviation of 3 and are coupled to qualitative terms as proposed by Lezak et al. (2012). Performance on tests that use cut‐offs for interpretation are scored as unimpaired (Z.R.'s score > cut‐off) or impaired (Z.R.'s score ≤ cut‐off).

However, as a visuospatial working memory deficit alone appeared an unlikely explanation for the severity of her navigation problems, we also assessed her navigation abilities in detail. The Virtual Tübingen (VT) test (see Section 2) was administered to measure Z.R.'s ability to learn new virtual routes (see Table 2) and her performance was compared to that of a matched control group. Her VT performance pattern indicates that she relies heavily on egocentric coding by remembering the sequence of turns. She has, however, problems with other egocentric (observer‐based) strategies, given her difficulties with forming associations between places and actions, and with remembering the order in which places occurred along the route. Her allocentric (environment‐based) knowledge of the route is also compromised as she has difficulties with metrical information, finds it hard to draw accurate maps and is unable to indicate the correct map out of four options. These results provide evidence for her difficulties with acquiring new routes related to both egocentric and allocentric strategies and show that she compensates for this inability through reliance on verbal route coding (e.g., left–right–left).

**Table 2:** Z.R.'s performance on the virtual Tübingen (VT) navigation test battery

VT subtask	Route A; January 18, 2016	Route B; April 25, 2016 a
Scene recognition	Total: 14/22 (64%)**	Total: 19/22 (86%)
	Targets: 4/11 (36%)	Targets: 8/11 (73%)
	Distractors: 10/11 (91%)	Distractors: 11/11 (100%)
Route continuation	5/11 (45%)	5/11 (45%)
Route sequence	6/7 (86%)	7/7 (100%)
Route order	8/33 (24%)	5/33 (15%)*
Route progression	61%**	60%**
Route distance	Not administered b	Not administered b
Distance estimation	400 m (correct: 400 m)	500 m (correct: 400 m)
Time estimation	300 s (correct: 210 s)	300 s (correct: 252 s)
Pointing to start	Not administered b	Not administered b
Pointing to end	Not administered b	Not administered b
Map drawing	2/11 (18%)	7/11 (64%)
Map recognition	Incorrect	Incorrect

*Note*. Scores marked with one (*) or two asterisks (**) indicate trend‐level impaired performance (*p* < 0.15, one‐sided) and impaired performance (*p* < 0.05, one‐sided), respectively. See Section 2 for an explanation.

a The VT navigation test battery was administered twice (using parallel versions), as Z.R. misunderstood the test instructions on the first administration (she indicated afterwards she had focused solely on the order of turns instead of memorizing as much as possible information from the route). Her patterns of performance were comparable across the two administrations for most subtasks, except for performance on the scene recognition subtask (impaired at the first assessment; intact at the second assessment). Her elevated performance on the Scene Recognition subtask is most likely related to a different attentional focus (i.e., she attended to the scenery more closely during the second administration). Although her performance on the Map Drawing subtask is higher on the second administration, it is debatable whether her score actually reflects better allocentric knowledge of the route. Despite a higher score on this subtask, she still was unable to recognize the correct map of the route among three distractors on the Map Recognition subtask.

b Z.R. was unable to understand the purpose of the subtask Route Distance. While Z.R., at the first administration, understood the purpose of the Pointing to Start subtask, it took her very long to provide a response on the first two trials. It was clear that she lacked knowledge about the direction of the starting point relative to the displayed scene and we decided to stop the subtask. The Pointing subtasks were skipped at the second administration of the VT test as well.

Lastly, we tested her knowledge of real‐world environments. We first assessed her landmark recognition ability by showing her pictures of famous landmarks. Most of them were accurately named (see Table 3). We then tested whether her difficulties with navigation were more prominent in environments she has never visited prior to the surgery, as she stated. We designed two equivalent tests to compare her environmental knowledge of a part of the city she grew up in (and still visits regularly) with that of the village she has lived in for the past 6 years (see Table 3). Her landmark recognition performance was sufficient for both environments (childhood city: 15/20 correct vs. current village: 17/20 correct). As a clinical observation, we noted that she relied on elaborate verbal reasoning to generate her responses on the location and route description tests. Although no healthy control data could be obtained for comparison due to the specificity of the environments, it appears that accurately indicating landmark locations is difficult for her for both environments. However, the relative differences in performance on the location tasks between her childhood city and her current village seem small (North–South axis: 9.1% vs. 14.5%; East–West axis: 12.0% vs. 16.0%). On the route description tasks, she correctly described three out of five routes for both environments. Overall, we established additional evidence for her difficulties with navigation based on these real‐world tests, but a substantial difference between knowledge of environments she has visited prior to and after the surgery was not confirmed.

**Table 3:** Z.R.'s performance on famous landmark recognition tasks and on two real‐world navigation tests based on her childhood city and current village

**Landmark recognition tasks** a	
Childhood city landmarks (city center)	8/10
Dutch landmarks	7/10
European landmarks	8.5/10
**Real‐world test childhood city**	
Landmarks^b^	Total: 15/20 correct (targets: 5/10, distractors: 10/10)
Locations^c^	North–south axis, average deviation from correct location: 9.1%
	East–west axis, average deviation from correct location: 12.0%
Route descriptions^d^	3/5
**Real‐world test current village**	
Landmarks^b^	Total: 17/20 correct (targets: 9/10, distractors: 8/10)
Locations^c^	North–south axis, average deviation from correct location: 14.5%
	East–west axis, average deviation from correct location: 16.0%
Route descriptions^d^	3/5

*Note*. Z.R. still travels on a regular basis to her childhood city to visit her father who lives there. Z.R. has lived in her current village for 6 years. She did not visit this area prior to the surgery. The stimuli presented in the tests were carefully matched between the two environments in terms of the functions of the landmarks (e.g., church, school, etc.) and distances.

a The scoring procedure for the landmark recognition tasks: 1 point was awarded for correct naming of the landmark; 0.5 point was given for a correct nonvisual description of the landmark.

b In this task, Z.R. was presented with 20 landmarks one by one (10 targets, 10 matched distractors) and we asked her to indicate whether each landmark was located in the target area.

c Z.R. was presented with maps of the environment in which only the outer sides were shown, while the center of the map was covered (full map: 24.8 cm × 14.3 cm; covered center: 19.7 cm × 10.2 cm). Z.R. was asked to indicate the location of the 10 target landmarks. Her performance was scored by calculating the percentage of deviation from the correct location, both on the North–South and East–West axes. A deviation of 1% equals 0.102 and 0.197 cm on the North–South and East–West axes respectively.

d Z.R. was asked to provide five detailed route descriptions between two landmarks in the environment. Route descriptions were considered correct if Z.R. described the correct order of turns at relevant decision points. Z.R. made three types of errors: describing an incorrect turn (1×), describing an incorrect decision point (1×), and taking a detour (2×).

Hence, we have provided evidence for severe difficulties with navigation in a patient who underwent a right anteromesial temporal lobectomy. While her performance on a neuropsychological assessment only showed a visuospatial working memory deficit, specific navigation tests (VT) clearly confirmed her difficulties with learning new routes related to both egocentric and allocentric strategies. In addition, we found deficits in her knowledge of landmark locations and the paths connecting these locations for two familiar environments. No clear evidence was found for a difference between test performance for environments visited prior to and after the surgery. It should, however, be noted that statistical comparisons to performance of healthy controls were not possible due to the specificity of the real‐world environments. The findings from the real‐world navigation tests should therefore be interpreted with some caution.

Still, the latter finding increases our knowledge about the hippocampal contribution to navigation ability. As stated above, previous case studies on patients with selective hippocampal damage (no temporal lobectomy patients, however) have reported mixed findings. Two case reports have suggested a time‐limited role of the hippocampus in spatial navigation, as their patients had difficulties only in novel and not familiar environments (Rusconi et al., 2008; Teng & Squire, 1999). Other case reports, however, have supported the idea that hippocampal involvement in navigation is permanent by showing navigation problems for both novel and familiar environments (Maguire et al., 2006; Rosenbaum et al., 2000, 2005). Our case report is rather in support of this latter position.

Another issue is the marked discrepancy between Z.R.'s intact performance on neuropsychological tasks for visuospatial abilities and episodic memory on the one hand and her impaired performance on the navigation tasks on the other hand. First, this might at least partly result from a difference in the spatial scale that these tests address. While the neuropsychological tasks measure small‐scale visuospatial skills (i.e., reaching space), our navigation tasks concern large‐scale visuospatial skills (i.e., navigational space). Striking dissociations between small and large‐scale visuospatial skills have previously been reported in brain‐injured patients (Piccardi, Iaria, Bianchini, Zompanti, & Guariglia, 2011) and these abilities are supported by partly different brain networks (Nemmi, Boccia, Piccardi, Galati, & Guariglia, 2013). The second explanation for the discrepancy relates to the two hippocampi being functionally lateralized. While the left hippocampus plays a central role in episodic memory, the right hippocampus is specialized in spatial processing for navigation (Burgess, Maguire, & O'Keefe, 2002). As only the right hippocampus is damaged in Z.R., she has difficulties with large‐scale spatial navigation tasks but not with the episodic memory tests. Given that Z.R. tends to remember routes as a sequence of left and right turns (verbal coding), it appears that she attempts to compensate for her difficulties with navigation by relying on the intact functioning of her left hippocampus.

One alternative but unlikely explanation that cannot be ruled out entirely is that Z.R.'s difficulties with navigation have a psychological origin. Long before the surgery, Z.R. has been diagnosed with a personality disorder due to an enduring and stable pattern of difficulties with establishing and maintaining social relationships. It can be hypothesized that she has once learned (after the surgery) that she raises the attention of other people when she has lost her way. For example, a neighbor has intensively helped her with recording written route descriptions, which might have reinforced Z.R. in displaying this behavior. However, it should be emphasized that Z.R. is now severely restricted in her mobility and autonomy.

Lastly, severe navigation impairment after right anteromesial temporal lobectomy appears to be a rarely described complication. The case report of Z.R. demonstrates that navigation impairment can have far‐reaching consequences. By describing this case, we intend to increase clinicians' awareness of the possibility of navigation problems as a complication of anteromesial temporal lobectomy, as this would help in gaining a better indication of its frequency of occurrence.

## 2 DETAILED METHODS

### 2.1 The virtual Tübingen test

The VT test is a virtual navigation test that can serve as a valid measure of real‐world navigation ability (Claessen, Visser‐Meily, de Rooij, Postma, & van der Ham, 2016). It comprises of a learning and a testing phase. Participants are first shown a movie of a short route through a virtual rendition of the German city Tübingen. The instruction is to remember as much as possible from the route. After having watched the route two times, the testing phase starts. Twelve subtasks (in fixed order) are used to assess the participant's knowledge of the route. *Scene Recognition* addresses the ability to discriminate between scenes that were and were not encountered in the route. *Route Continuation* requires participants to indicate the direction in which the route continued at randomly presented decision points. *Route Sequence* tests whether participants have remembered the sequence of turns taken. *Route Order* verifies the participants' ability to arrange scenes according to the order in which they occurred in the route. In the *Route Progression* subtask, participants are asked to indicate the position of scenes in the route on a line (representing the total distance of the route). In the subtask *Route Distance*, participants are presented with a pair of scenes and have to indicate the distance between them on a line that represents total route distance. In the *Distance Estimation* and *Time Estimation* subtasks, participants are asked to provide estimates of the distance and duration of the route. The *Pointing to Start* and *Pointing to End* subtasks require participants, imagining standing at particular scenes, to indicate the start and end point of the route using a rotational device. Participants attempt to draw the route on a map of VT in the *Route Drawing* subtask. Lastly, in the *Map Recognition* subtask, participants are asked to choose the map that correctly displays the route, which is presented along with three distractors. A more extensive description of the VT test can be found in the paper by Claessen et al. (2016).

### 2.2 Statistical analyses

Z.R.'s scores on the VT test were compared to those of a healthy control group comprising 11 women, *M*_age_ = 62.1 (Z.R. = 66), *M*_educational level_ = 5.6 (Z.R. = 5, possible range: 1–7). Statistical comparisons were made using the Bayesian approach for single case studies (Crawford & Garthwaite, 2007). The computer program “SingleBayes_ES.exe” was used with the 95% credible interval setting “one‐sided lower”. This program requires entering the mean, standard deviation, and sample size of the control group and the test score of the patient for each VT subtest.

#### Informed consent

Z.R. has provided informed consent and agreed with publication of the study.
